# Supplementary material for: COVID-19 patient profiles over four waves in Barcelona metropolitan area: A clustering approach
Source: PLoS One. 2024 May 7;19(5):e0302461. doi: 10.1371/journal.pone.0302461 (PMC11075871; doi:10.1371/journal.pone.0302461)
Supplement: S1 File — (DOCX) [file pone.0302461.s001.docx]

**Supplemental Online Content**

**COVID-19 patient profiles over four waves in Barcelona metropolitan area: A clustering approach**

**eTable 1**. Number of patients included in the study broken down by health consortiums and wave number.

| **Center** | **Wave 1** | **Wave 2** | **Wave 3** | **Wave 4** |
| --- | --- | --- | --- | --- |
| Hospital Universitari de Bellvitge | 805 | 319 | 380 | 559 |
| Consorci Sanitari de l'Alt Penedès i Garraf | 165 | 167 | 297 | 228 |
| Hospital de Viladecans | 156 | 52 |  |  |
| Hospital de Sant Boi de Llobregat | 531 | 159 |  |  |

**eTable2.** Data description of the variables per wave used to determine groups.

|  | **Wave 1**  **N = 1657** | **Wave 2**  **N = 697** | **Wave 3**  **N = 677** | **Wave 4**  **N = 787** |
| --- | --- | --- | --- | --- |
| *Demographic characteristics and comorbidities* | | | | |
| **Age**, Median [Q1; Q3] | 66.0 [54, 76] | 65.0 [55, 77] | 66.0 [54, 77] | 56.0 [40, 73] |
| **Gender** - **Women**, N (%) | 658 (39.7) | 256 (36.7) | 289 (42.7) | 293 (37.2) |
| **Smoker**, N (%): | 453 (27.3) | 190 (27.2) | 220 (32.5) | 205 (26.0) |
| **Alcohol consumption**, N (%) | 55 (3.3) | 38 (5.5) | 31 (4.6) | 32 (4.1) |
| **Charlson Index**, Mean (SD) | 3.3 (2.5) | 3.5 (2.7) | 3.5 (2.5) | 2.5 (2.6) |
| **Obesity (BMI > 30**), N (%) | 506 (36.6) | 238 (34.1) | 280 (41.4) | 275 (34.9) |
| **Diabetes mellitus**, N (%) | 408 (24.6) | 160 (23.0) | 161 (23.8) | 138 (17.5) |
| **Arthromyalgias**, N (%) | 560 (33.8) | 201 (28.8) | 186 (27.5) | 215 (27.3) |
| **Dementia**, N (%) | 99 (6.0) | 52 (7.5) | 41 (6.1) | 40 (5.1) |
| **COPD**, N (%) | 315 (19.0) | 140 (20.1) | 151 (22.3) | 157 (19.9) |
| **Degenerative neurological**, N (%) **disease**, N (%) | 42 (2.5) | 29 (4.2) | 12 (1.8) | 6 (0.8) |
| **Peripheral vascular disease**, N (%) | 63 (3.8) | 48 (6.9) | 52 (7.7) | 23 (2.9) |
| **Abdominal pain**, N (%) | 102 (6.2) | 28 (4.0) | 22 (3.2) | 44 (5.6) |
| **Heart failure**, N (%) | 121 (7.3) | 52 (7.5) | 53 (7.8) | 58 (7.4) |
| **Mild renal insufficiency**, N (%) | 89 (5.4) | 40 (5.7) | 28 (4.1) | 10 (1.3) |
| **TIA**, N (%) | 107 (6.5) | 41 (5.9) | 43 (6.4) | 35 (4.4) |
| **Hypertension**, N (%) | 843 (50.9) | 352 (50.5) | 363 (53.6) | 281 (35.7) |
| **Vaccinated (partial or full)**, N (%) |  |  |  | 351 (44.6) |
| **Vaccination dose received**, N (%) |  |  |  |  |
| No vaccine |  |  |  | 436 (55.4) |
| Partial regimen |  |  |  | 59 (7.5) |
| Full regimen |  |  |  | 292 (37.1) |
| *Vital signs, severity scores, and clinical analytics at hospital admission* | | | | |
| **Temperature**, N (%) |  |  |  |  |
| **FiO2**, Mean (SD) | 28.2 (18.9) | 31.1 (21.2) | 33.0 (22.4) | 32.2 (20.3) |
| **SatO2**, Mean (SD) | 93.6 (6.8) | 94.2 (4.4) | 95.0 (4.4) | 95.3 (3.3) |
| **SatO2/FiO2**, Mean (SD) | 396.6 (107.0) | 376.3 (116.4) | 365.4 (123.9) | 363.5 (117.3) |
| **ROX index**, Mean (SD) | 19.5 (7.7) | 18.4 (7.5) | 18.3 (7.7) | 18.5 (7.3) |
| **Systolic blood pressure**, Mean (SD) | 131.0 (21.2) | 131.8 (21.6) | 133.7 (20.3) | 127.1 (20.2) |
| **Diastolic blood pressure**, Mean (SD) | 73.9 (14.1) | 73.9 (12.7) | 76.9 (12.8) | 73.4 (11.5) |
| **Heart rate**, Mean (SD) | 90.5 (18.7) | 87.6 (18.4) | 86.7 (16.3) | 85.5 (17.1) |
| **Respiratory rate**, Mean (SD) | 22.3 (6.6) | 22.2 (5.8) | 21.5 (5.4) | 20.9 (4.9) |
| **MULBSTA**, Mean (SD) | 8.1 (3.4) | 8.6 (3.5) | 9.0 (3.6) | 7.9 (3.5) |
| **PSI group**, N (%) |  |  |  |  |
| 1 | 781 (47.1) | 290 (41.6) | 329 (48.6) | 414 (52.6) |
| 2 | 361 (21.8) | 171 (24.5) | 148 (21.9) | 135 (17.2) |
| 3 | 383 (23.1) | 166 (23.9) | 158 (23.3) | 179 (22.7) |
| 4 | 132 (8.0) | 70 (10.0) | 42 (6.2) | 59 (7.5) |
| **CURB-65 group**, N (%) |  |  |  |  |
| Low risk | 1079 (65.1) | 424 (60.8) | 429 (63.4) | 563 (71.6) |
| Intermediate risk | 375 (22.6) | 178 (25.6) | 182 (26.9) | 167 (21.2) |
| High risk | 203 (12.3) | 95 (13.6) | 66 (9.7) | 57 (7.2) |
| **Severe Pneumonia**, N (%) | 677 (40.9) | 334 (47.9) | 333 (49.2) | 350 (44.5) |
| **Angiotensin receptor antagonists**, N (%) | 241 (14.5) | 87 (12.5) | 93 (13.7) | 85 (10.8) |
| **Angiotensin-converting enzyme**, N (%) | 351 (21.2) | 155 (22.2) | 152 (22.5) | 124 (15.8) |
| **Lymphosits (mil/mm³)**, Mean (SD) | 1.1 (1.3) | 1.0 (0.8) | 1.2 (2.6) | 1.1 (1.2) |
| **Neutrophils** **(mil/mm³)**, Mean (SD) | 5.8 (3.4) | 6.0 (3.8) | 5.7 (3.5) | 5.4 (3.5) |

BMI: Body Mass Index, PSI: Pneumonia Severity Index, MuLBSTA: Viral Pneumonia Mortality Score; TIA: Stroke/transient ischemic attack; CURB-65: Pneumonia severity score^38^

**eTable 3**. Comparison of demographic characteristics, comorbidities, vital signs and severity scores at hospital admission between clusters for wave 2 (October 1st - November 31st, 2020). The table only shows the statistically significant variables^1^.

| **Wave 2 (3 clusters)** | **Cluster 1 (n_w2_=242, 34.7%)** | **Cluster 2 (n_w2_=241, 34.6%)** | **Cluster 3 (n_w2_=214, 30.7%)** |
| --- | --- | --- | --- |
| *Demographic characteristics and comorbidities* | | | |
| **Age**, Median [Q1; Q3] | 56.5 [48.0, 65.0] | 74.0 [63.0, 83.0] | 69.9 [59.0, 79.0] |
| **Gender** - **Women**, N (%) | 84 (34.7) | 99 (41.1) | 73 (34.1) |
| **Obesity (BMI > 30**), N (%) | 69 (28.5) | 81 (33.6) | 88 (41.1) |
| **Charlson Index**, Mean (SD) | 1.9 (1.8) | 4.7 (2.8) | 3.9 (2.5) |
| **Dementia**, N (%) | 9 (3.7) | 30 (12.4) | 13 (6.1) |
| **Heart failure**, N (%) | 2 (0.8) | 30 (12.4) | 20 (9.3) |
| **Hypertension**, N (%) | 0 (0.0) | 241 (100.0) | 111 (51.9) |
| **TIA**, N (%) | 1 (0.4) | 26 (10.8) | 14 (6.5) |
| *Vital signs, severity scores, and clinical analytics at hospital admission* | | | |
| **FiO_2_**, Mean (SD) | 21.4 (1.4) | 21.2 (1.0) | 53.3 (7.4) |
| **SatO_2_/FiO_2_**, Mean (SD) | 442.0 (27.6) | 441.0 (28.0) | 229.0 (55.0) |
| **SatO_2_**, Mean (SD) | 94.5 (3.7) | 93.3 (5.1) | 94.9 (4.1) |
| **ROX index**, Mean (SD) | 22.4 (4.4) | 21.9 (5.0) | 9.9 (5.5) |
| **PSI group**, N (%) |  |  |  |
| 1 | 168 (69.4) | 72 (29.9) | 50 (23.3) |
| 2 | 51 (21.5) | 60 (24.9) | 59 (27.6) |
| 3 | 21 (8.7) | 80 (33.2) | 65 (30.4) |
| 4 | 1 (0.4) | 29 (12.0) | 40 (18.7) |
| **CURB-65 group**, N (%) |  |  |  |
| Low risk | 209 (86.3) | 114 (47.3) | 101 (47.2) |
| Intermediate risk | 21 (11.2) | 88 (36.5) | 39 (29.4) |
| High risk | 101 (2.5) | 63 (16.2) | 50 (23.4) |
| **MuLBSTA**, Mean (SD) | 6.4 (3.0) | 9.7 (3.1) | 10.0 (3.3) |
| **Severe Pneumonia**, N (%) | 58 (24.0) | 79 (32.8) | 191 (89.3) |
| **Respiratory rate**, Mean (SD) | 20.5 (4.7) | 21.3 (5.4) | 25.1 (6.4) |
| **Lymphosits (mil/mm³)**, Mean (SD) | 1.1 (1.0) | 1.1 (0.6 | 0.9 (0.8) |
| **Angiotensin-converting enzyme**, N (%) | 3 (1.2) | 99 (41.1) | 53 (24.8) |

1. Based on Mann-Whitney U test for numerical variables and Chi-squared tests for categorical variables. Gender was not significant but it was included for demographic interest; 2. BMI: Body Mass Index, PSI: Pneumonia Severity Index, COPD: Chronic pulmonary pathology, ROX: Ratio of oxygen saturation, MuLBSTA: Viral Pneumonia Mortality Score; TIA: Stroke/transient ischemic attack; CURB-65: Pneumonia severity score^38^

**eTable 4**. Comparison of demographic characteristics, comorbidities, vital signs and severity scores at hospital admission between clusters for wave 3 (January 1st - February 28th, 2021). The table only shows the statistically significant variables^1^.

| **Wave 3 (2 clusters)** | **Cluster 1**  **(n_w3_=221, 32.6%)** | **Cluster 2**  **(n_w3_=456, 67.4%)** |
| --- | --- | --- |
| *Demographic characteristics and comorbidities* | | |
| **Age**, Median [Q1; Q3] | 70.0 [59.0,77.0] | 64.0 [53.0,76.0] |
| **Gender** - **Women**, N (%) | 85 (38.5) | 204 (44.7) |
| **Obesity (BMI > 30**), N (%) | 103 (46.6) | 177 (38.8) |
| **Charlson Index**, Mean (SD) | 4.1 (2.6) | 3.2 (2.4) |
| **Hypertension**, N (%) | 132 (59.7) | 231 (50.8) |
| **TIA**, N (%) | 21 (9.5) | 22 (4.8) |
| **Arthromyalgias**, N (%) | 45 (20.4) | 141 (30.9) |
| *Vital signs, severity scores, and clinical analytics at hospital admission* | | |
| **FiO_2_**, Mean (SD) | 56.3 (16.8) | 21.8 (2.1) |
| **SatO_2_/FiO_2_**, Mean (SD) | 214.0 (82.0) | 439.0 (36.5) |
| **ROX index**, Mean (SD) | 9.7 (5.4) | 22.5 (4.5) |
| **PSI group**, N (%) |  |  |
| 1 | 64 (29.0) | 265 (58.1) |
| 2 | 59 (26.7) | 89 (19.5) |
| 3 | 67 (30.3) | 91 (20.0) |
| 4 | 31 (14.0) | 11 (2.4) |
| **CURB-65 group**, N (%) |  |  |
| Low risk | 108 (48.9) | 321 (70.4) |
| Intermediate risk | 76 (34.4) | 106 (23.2) |
| High risk | 37 (16.7) | 29 (6.4) |
| **MuLBSTA**, Mean (SD) | 10.7 (3.1) | 8.1 (3.5) |
| **Severe Pneumonia**, N (%) | 211 (95.5) | 122 (26.8) |
| **Respiratory rate**, Mean (SD) | 23.9 (6.4) | 20.3 (4.5) |
| **Lymphosits (mil/mm³)**, Mean (SD) | 0.8 (0.6) | 1.3 (3.1) |

1. Based on Mann-Whitney U test for numerical variables and Chi-squared tests for categorical variables. Gender was not significant but it was included for demographic interest; 2. BMI: Body Mass Index, PSI: Pneumonia Severity Index, COPD: Chronic pulmonary pathology, ROX: Ratio of oxygen saturation, MuLBSTA: Viral Pneumonia Mortality Score; CURB-65: Pneumonia severity score^38^

**eFigure1.** Barcelona health metropolitan south region.


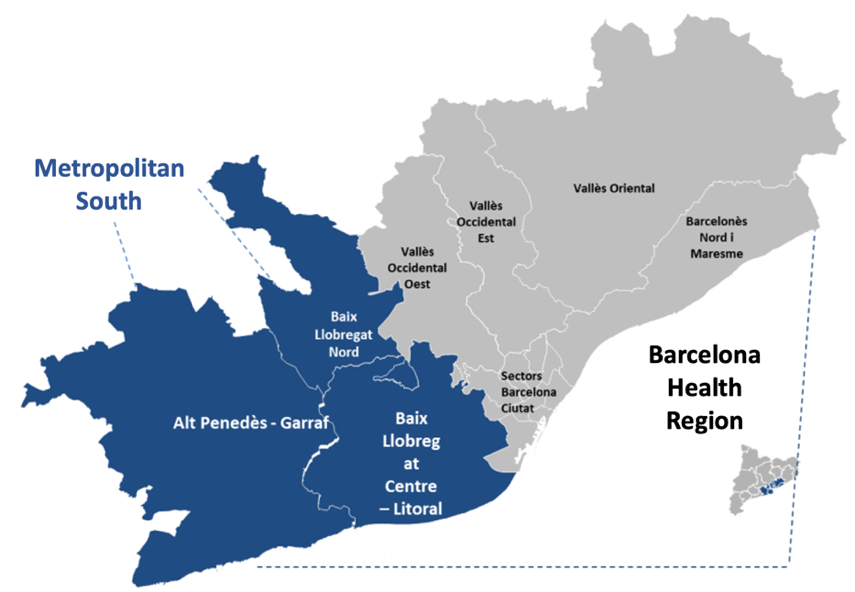


**eFigure2.** Prediction strength values for the KAMILA clustering in the four waves: wave 1 (top left), wave 2 (top right), wave 3 (bottom left), and wave 4 (bottom right). Prediction strength values are plotted against the number of clusters (2 to 10), with error bars denoting plus or minus one standard error. The horizontal dotted line at (y = 0.8 for waves 1-3, and y = 0.65 for wave 4) denotes the default threshold for determining the number of clusters.


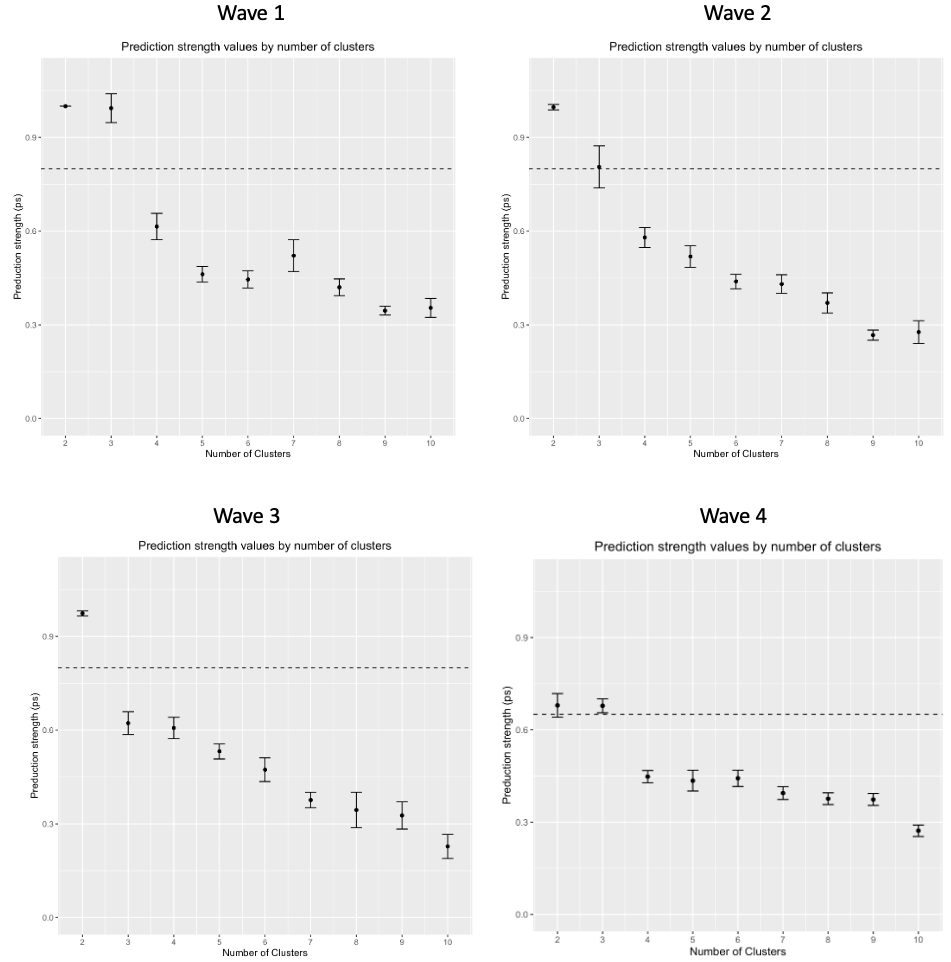


**eFigure3.** Results of clustering the of COVID-19 data set in eight third level hospitals in the Barcelona metropolitan south region using KAMILA for four waves (the first wave included COVID-19-hospitalized patients between March 1st and April 15th, 2020; the second wave, from October 1st to November 31st, 2020; third, from January 1st to February 28th, 2021; and fourth, from July 1st to August 31st, 2021). The clusters are plotted using the first two principal components of data. Cluster number is depicted by color.


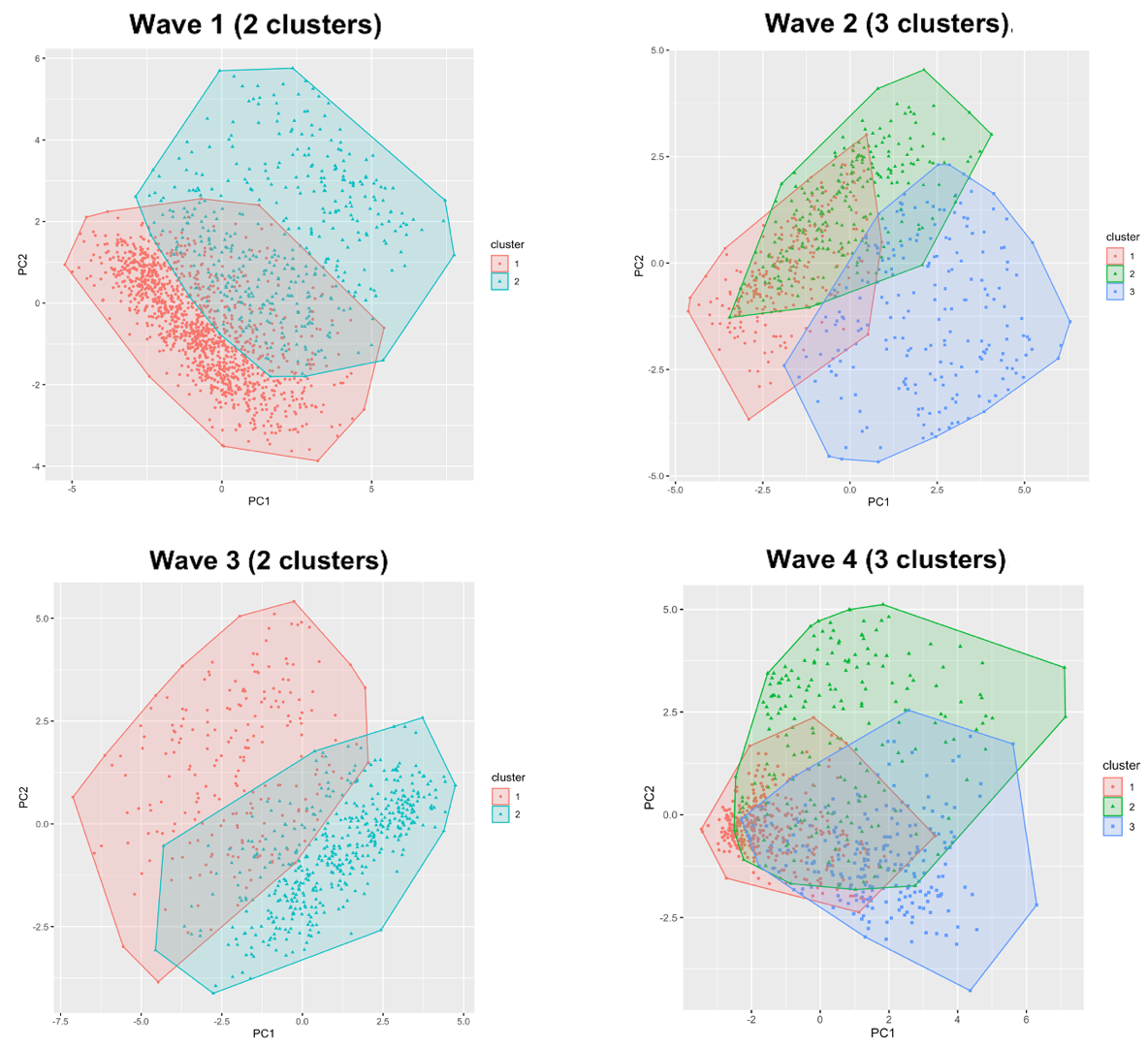


**eFigure4.** Comparison of sociodemographic and clinical characteristics^1^ between KAMILA clusters for each wave: wave 1 (top left), wave 2 (top right), wave 3 (bottom left), and wave 4 (bottom right). The figure only depicts the statistically significant variables^2^.


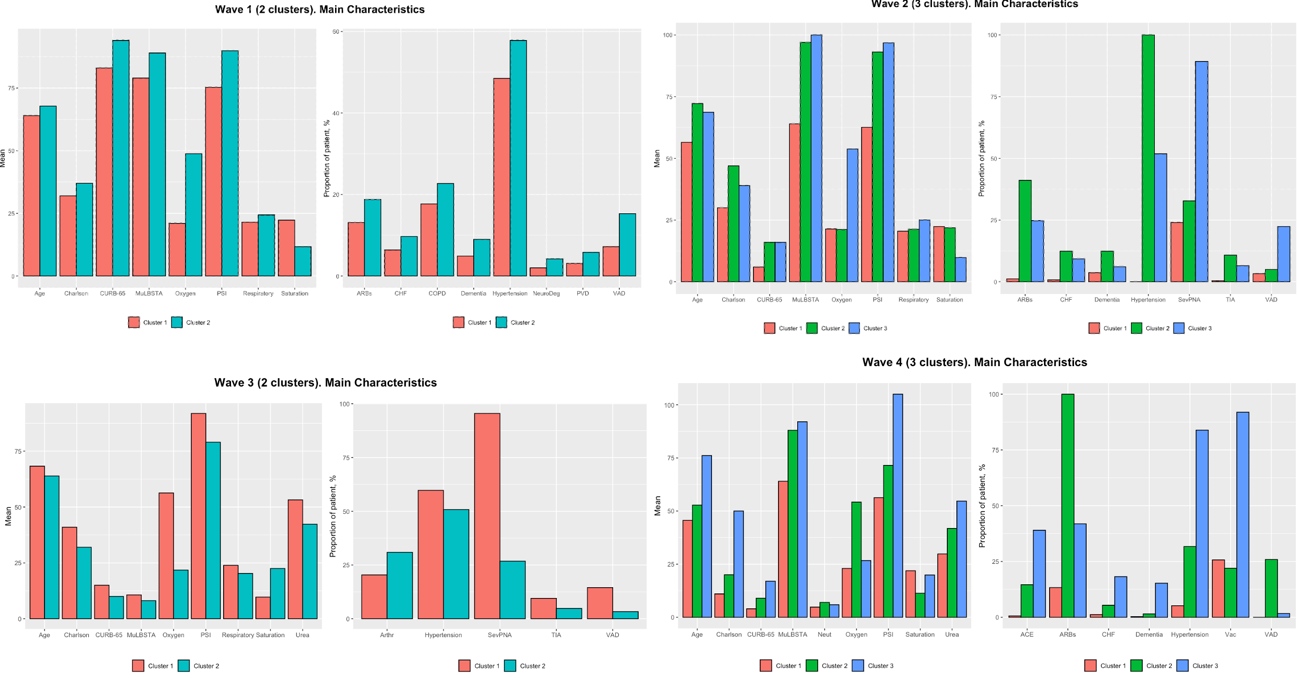


1. Age: age of the patient at hospital admission; Charlson: Charlson comorbidity index; CURB-65: Pneumonia severity score; MuLBSTA: Viral pneumonia mortality score; Oxygen: Fraction of inspired oxygen; PSI: Pneumonia Severity Index; Respiratory: Respiratory frequency; Saturation: Ratio of oxygen saturation; Neut: Neutrophils; ARBs: Angiotensin receptor antagonists; CHF: Heart failure; COPD: Chronic pulmonary pathology; NeuroDeg: Degenerative neurological disease; PVD: Peripheral vascular disease; VAD: Vasoactive drugs; SevPNA: Severe Pneumonia; TIA: Stroke/transient ischemic attack; Arthr: Arthromyalgia; ACE: Angiotensin-converting enzyme; Vac: Vaccinated (partial or full);

2.Based on Mann-Whitney U test for numerical variables and Chi-squared tests for categorical variables

This supplemental material has been provided by the authors to give readers additional information about their work.
