# Supplementary material for: COVID-19 patient profiles over four waves in Barcelona metropolitan area: A clustering approach
Source: PLoS One. 2024 May 7;19(5):e0302461. doi: 10.1371/journal.pone.0302461 (PMC11075871; doi:10.1371/journal.pone.0302461)
Supplement: Attachment — Submitted filename: Response_to_Reviewers_R1.docx [file pone.0302461.s002.docx]

Response to reviewers' comments on the paper “COVID-19 patient profiles over four waves in Barcelona metropolitan area: A clustering approach”

April, 21st 2023

**General Comments to Editor**

We are very grateful for the opportunity to revise our manuscript “COVID-19 patient profiles over four waves in Barcelona metropolitan area: A clustering approach” (Manuscript Number: PONE-D-22-31704) for PLOS ONE. We carefully considered the reviewers' comments, and below we explain how we have revised the paper based on those comments and recommendations. Furthermore, we hope that those revisions improve the paper such that you and the reviewers now deem it worthy of publication in PLOS ONE. Below, we offer detailed responses to the reviewers' comments (written in blue). For the revised manuscript, all changes are tracked to ease their identification.

Our revised paper focused on the following key points:

- We amended the description of the funding information and the data availability, following the Journal Requirements listed by the Academic Editor.
- We rewrote quite a few parts of the article following reviewers' comments and suggestions to make it clearer.
- We moved one table (current Table 3) from supplementary material to the main body, which makes the manuscript more comprehensive.
- We extended the state-of-art, adding three references.
- We added a better-quality image of Figure 1.
- We thoroughly proofread the whole manuscript.

**Response to Academic Editor (Dr. Bhaskar)**

1. Please ensure that your manuscript meets PLOS ONE's style requirements, including those for file naming. The PLOS ONE style templates can be found at

https://journals.plos.org/plosone/s/file?id=wjVg/PLOSOne_formatting_sample_main_body.pdf and

https://journals.plos.org/plosone/s/file?id=ba62/PLOSOne_formatting_sample_title_authors_affiliations.pdf

Thank you for the style templates. We believe that we have followed the PLOS ONE’s style requirements.

2. Please amend your current ethics statement to address the following concerns: a) Did participants provide their written or verbal informed consent to participate in this study? b) If consent was verbal, please explain it) why written consent was not obtained, ii) how you documented participant consent, and iii) whether the ethics committees/IRB approved this consent procedure.

We have amended it in the revised version of the manuscript. Now it reads as follows in the Methods section):

*“The study was approved by the ethics committee (CEIm Hospital Universitari de Bellvitge) in accordance with Spanish legislation and was performed in accordance with the Helsinki Declaration of 1964. The need for patient informed consent was waived by the ethics committee.”*

3. Please amend your current ethics statement to include the full name of all ethics committees/institutional review boards that approved your specific study.

The ethics committee who approved the project was “CEIm Hospital Universitari de Bellvitge”. We amended it in the ethics statement of the revised manuscript:

*“The study was approved by the ethics committee (CEIm Hospital Universitari de Bellvitge) in accordance with Spanish legislation and was performed in accordance with the Helsinki Declaration of 1964. The need for patient informed consent was waived by the ethics committee.”*

4. Thank you for stating the Acknowledgments Section of your manuscript. However, funding information should not appear in the Acknowledgments section or other areas of your manuscript. We will only publish funding information present in the Funding Statement section of the online submission form.

Please remove any funding-related text from the manuscript and let us know how you would like to update your Funding Statement. Please include your amended statements within your cover letter; we will change the online submission form on your behalf.

We removed from the revised version of the manuscript and included the following amended version of the funding statement in the cover letter:

*DF, NP, and GM have been supported by l’Agència de Gestió d'Ajuts Universitaris i de Recerca (AGAUR) de la Generalitat de Catalunya (Spain) [2020PANDE00148] (https://agaur.gencat.cat/en/inici/index.html). DF and NP have been supported by the Ministerio de Ciencia e Innovación (Spain) [PID2019-104830RB-I00/ DOI (AEI): 10.13039/501100011033] (*[*https://www.aei.gob.es/en/announcements/announcements-finder/proyectos-idi-2019-modalidades-retos-investigacion-generacion*](https://www.aei.gob.es/en/announcements/announcements-finder/proyectos-idi-2019-modalidades-retos-investigacion-generacion)*) and by grant 2021 SGR 01421 (GRBIO) administrated by the Departament de Recerca i Universitats de la Generalitat de Catalunya (Spain) (*[*https://tauler.seu.cat/pagDetall.do?idEdicte=419801&idens=1*](https://tauler.seu.cat/pagDetall.do?idEdicte=419801&idens=1)*). The funders had no role in study design, data collection and analysis, decision to publish, or preparation of the manuscript.*

5. We note that you have indicated that data from this study are available upon request. PLOS only allows data to be available upon request if there are legal or ethical restrictions on sharing data publicly. In your revised cover letter, please address the following prompts:

a) If there are ethical or legal restrictions on sharing a de-identified data set, please explain them in detail (e.g., data contain potentially sensitive information, data are owned by a third-party organisation, etc.) and who has imposed them (e.g., an ethics committee). Please also provide contact information for a data access committee, ethics committee, or other institutional body to which data requests may be sent.

b) If there are no restrictions, please upload the minimal anonymized data set necessary to replicate your study findings as either Supporting Information files or to a stable, public repository and provide us with the relevant URLs, DOIs, or accession numbers. For a list of acceptable repositories,

We will update your Data Availability statement on your behalf to reflect the information you provide.

The Ethics Committee of Bellvitge Hospital grants access to data on a study-by-study basis. Additionally, the DIVINE research group has agreed that data will only be shared upon request and after evaluation of the purpose and objectives of the study. The DIVINE research group can be contacted via e-mail ([grbio@grbio.eu](mailto:grbio@grbio.eu)) and the Ethics Committee of Bellvitge Hospital can be also contacted via e-mail ([presidenciaceic@bellvitgehospital.cat](mailto:presidenciaceic@bellvitgehospital.cat)).

We have included this information in the cover letter.

6. One of the noted authors is a group or consortium DIVINE project. In addition to naming the author group, please list the individual authors and affiliations within this group in the acknowledgments section of your manuscript. Please also indicate clearly a lead author for this group along with a contact email address.’

We included the requested information in the acknowledgments section.

The DIVINE research team is composed by (apart from the three authors of this article): Gabriela Abelenda Alonso^1,2^; Mireia Besalú^2^; Jordi Carratalà^1,3,4,5^; Erik Cobo^6^; Jordi Cortés^6^; Leire Garmendia^6^; Guadalupe Gómez-Melis^6^; Carlota Gudiol^1^; Pilar Heureu^1^, Natàlia Pallarès^7^; Klaus Langohr^6^; Xavier Piulachs^6^; Alexander Rombauts^1,2^; Cristian Tebé^7^; Sebastián Videla^8,9^

^1^Department of Infectious Diseases, Bellvitge University Hospital, Barcelona, Spain.

^2^Bellvitge Biomedical Research Institute (IDIBELL), Barcelona, Spain.

^3^Department of Clinical Sciences, School of Medicine and Health Sciences, University of Barcelona, Barcelona, Spain.

^4^Bellvitge Biomedical Research Institute (IDIBELL), Barcelona, Spain.

^5^CIBERINFEC, Instituto de Salud Carlos III, Madrid, Spain.

^6^Department of Statistics and Operations Research, Universitat Politècnica de Catalunya/Barcelonatech, Barcelona, Spain.

^7^Biostatistics Unit of the Bellvitge Biomedical Research Institute (IDIBELL), L'Hospitalet de Llobregat, Avinguda de la Granvia de l'Hospitalet, 199, 08908, Barcelona, Spain.

^8^Department of Clinical Pharmacology, Bellvitge University Hospital, Barcelona, Spain.

^9^Department of Pathology and Experimental Therapeutics, School of Medicine and Health Sciences, University of Barcelona, Barcelona, Spain.

The leaders (and e-mail addresses) of this group are Cristian Tebé ([ctebe@idibell.cat](mailto:ctebe@idibell.cat)) and Guadalupe Gómez-Melis ([lupe.gomez@upc.edu](mailto:lupe.gomez@upc.edu)).

7. Your ethics statement should only appear in the Methods section of your manuscript. If your ethics statement is written in any section besides the Methods, please move it to the Methods section and delete it from any other section. Please ensure that your ethics statement is included in your manuscript, as the ethics statement entered into the online submission form will not be published alongside your manuscript.

We moved into the Methods section.

**Response to Reviewers**

**Reviewer #1**

In this article, the Authors report the results of a prospective multicentre cohort study based on patient clusterization during four COVID-19 waves within the southern Barcelona metropolitan area. Their aim is to identify different patient profiles and to relate them to the severity degrees of the outcomes. The study was conducted appropriately, with a methodology employing a clustering classification based on multistep procedure. However, description of the steps undertaken was lacking, with grammatical and syntax errors that made the reading difficult to follow. Furthermore, we recommend extensive English language editing for the whole document. In the list below, Authors can find a number of points that specifically need to be addressed in order to improve the quality of the article.

We have taken the reviewer's feedback into consideration and have made significant revisions to the manuscript following the reviewer's comments and suggestions. In order to enhance its comprehensiveness, we thoroughly proofread the whole manuscript (all changes can be tracked throughout the revised version of the manuscript). Additionally, we have provided a detailed explanation of the changes made in response to the reviewer's comments.

Abstract
− Objectives: syntax and meaning of the sentence are not clear.

We have revised the sentence to better convey the aim of our study. The sentence reads now as follows:

*“****Objectives:*** *Identifying profiles of hospitalized COVID-19 patients and explore their association with different degrees of severity of COVID-19 outcomes (i.e. in-hospital mortality, ICU assistance, and invasive mechanical ventilation). The findings of this study could inform the development of multiple care intervention strategies to improve patient outcomes.”*

− Methods: the phrase “level of obesity” is not exact. In both table 1 and 2 it is only specified the number of patients with a BMI score above 30 and not the level of obesity. Use “number of obese patients”, instead.

Introduction

Thank you for this suggestion. We changed “level of obesity” for “number of obese patients” throughout the revised version of the manuscript as it appeared four times.

− In the first sentence it is better to use “a.k.a” in the extended form “also known as”.

We replaced the abbreviation “a.k.a.” by “also known as” throughout the revised version of the manuscript as it appeared twice.

Methods
a) Setting and participants:

o Was the informed consent necessary? Did the Authors acquired it?

We have amended it in the revised version of the manuscript. Now it reads as follows:

*“The study was approved by the ethics committee of all participating institutions in accordance with Spanish legislation and was performed in accordance with the Helsinki Declaration of 1964. The need for patient informed consent was waived by each ethics committee.”*

o The sentence ”All patients were adults (>18 years old) and admitted with PCR-proven SARS-CoV-2 infection for at least 48 hours” is not clear: did the PCR test has to become positive in the 48 hours before or following admission?

Thank you for your question as it needs to be clarified. All participants had a positive PCR-proven test before the admission. We rewrote the sentence as follows:

*“All patients were adults (>18 years old) and were admitted with a PCR-proven SARS-CoV-2 infection that occurred before admission, with a maximum of 48 hours between the positive test and admission”.*

b) Analytic process:

1. Collecting and formatting of study data: in the second sentence it is not specified who did in particular fill the questionnaires. Did the Authors or other physicians do it?

We fixed this omission. We rewrote the sentence as follows:

*“The questionnaires were designed by joint agreement of the clinical and statistician teams and filled in by physicians in each hospital”*

Interpretation of cluster profiles:

− In the sentence “…we shared our analytic results with the physicians to define…” which are the physicians the Authors are referring to? How were the distinct profiles defined? Was there consensus between the MDs in the definitions? In the last sentence it is not clear what the Authors are trying to say.

Thank you for this set of questions, as we realised we were not clear in the manuscript. We wrote the following text in the revised version of the manuscript:

*“Thirdly, we presented the clustering structures resulting from the statistical analysis to physicians who are part of the DIVINE research team and worked with them to define clinically meaningful patient profiles. During this procedure, we discussed the medical interpretation and contextualization of the results, and reached a consensus on the definitions. ”*

Results
− KAMILA Results:

o The sentence “Generally speaking, the more determined variables for the three first waves to find the clusters were patient’s age and level of obesity, the number of comorbidities, oxygen support requirement, and various severity scores as SaFi illness, which is defined as the ratio between the oxygen saturation and the fraction of inspired oxygen” is ambiguous, so it is necessary to review it.

Thank you for your comment regarding the sentence on the variables that were important in determining the clusters for the first three waves. We agree that the previous sentence could have been clearer, and we have revised it as follows:

*“Generally speaking, the variables that were found to be most important in determining the clusters for the first three waves included the patient's age, the number of obese patients, the number of comorbidities, the requirement for oxygen support, and various severity scores such as SaFi illness^37,38^. SaFi illness is defined as the ratio between the oxygen saturation and the fraction of inspired oxygen. (i.e., SatO_2_/FiO_2_). For the last wave, the vaccination dose that the patient has received is the most determining variable.”*

We hope that this revised sentence is now more clear.

o In the same sentence as above, it’s more appropriate the use of “i.e” instead of “a.k.a”.

Changed.

− Patient Profiles:

o When referring to age or ages use “lower” instead of “smaller”.

Changed throughout the section.

o In the last sentence of the first paragraph “degrees of obesity” is not correct (see Abstract in this review).

Changed. It reads now as follows:

*“It also includes more men and a greater number of obese patients. ”*

o The 2nd and 3rd paragraph need a significant language and syntax editing.

We have applied the language and syntax editing and now those two paragraphs read as follows:

*“In Wave 2 (see eTable 4), the patients were divided into three clusters. Cluster 3 had a higher percentage of men, more obese patients, and presented worse prognostic indicators (see Figure 1). Cluster 1 included younger patients with fewer comorbidities, while the opposite was found for patients in Cluster 2.*

*For patients in Wave 3 (see eTable 5), two clusters were identified. There were no significant differences in the number of comorbidities (Charlson index) between the clusters, but Cluster 1 contained patients with worse health outcomes, lower SatO_2_/FiO_2_ levels, older age, higher pneumonia scores, and more severe pneumonia symptoms.”*

o In the 4th paragraph the sentence “Between the two non-vaccinated….combined with larger ages” has multiple issues:

Thank you for pointing them out.

♣ Is better to use “less-vaccinated” than “non-vaccinated” because in cluster 1 and 2 the vaccination rate is low but not null.

Changed.

♣ The full regimen vaccination rate is actually lower in the first cluster (15.3%) and not in the 2nd (16.6 %).

Changed.

♣ “Larger ages” is not correct, better to use “a wider age’s range”.

Changed.

o In the sentence “The elderly patients (cluster 3)….the survivors of previous waves.” it’s not clear how the Authors draw these conclusions. Have only the patients in Cluster 3 survived the previous COVID-19 waves? Did these patients catch SARS-Cov-2 during previous waves?

Thank you for providing your valuable feedback. We apologize for any confusion caused by the statement regarding elderly patients in Cluster 3. We want to clarify that this conclusion was not based on specific data analysis but drawn from discussions with the physicians on the DIVINE research team. As the patients in Cluster 3 are mostly elderly, and this age group had a higher rate of deaths during the first waves, we assumed that they might have been survivors of the previous waves. To better convey our intended meaning, we have revised the sentence as follows:

*“The elderly patients (Cluster 3), despite having a larger number of comorbidities, are not labeled as the worst prognostic group as they received full-regimen vaccine and could have potentially survived the previous waves. In the current wave (July 1st - August 31st, 2021), they may have also received full-regimen vaccination, which could have contributed to their improved prognosis.”*

We hope that this clarifies any confusion.

o eTable 3 should be added to the main article because it is useful to better understand the different outcomes in the different clusters.

We agree with the reviewer and have added it into the main body of the article. We have also renumbered all tables to be consistent.

Discussion
− The meaning of the last sentence of the 1st paragraph, as highlighted before in the results, is not clear. Does it mean that the patients in the other clusters didn’t survive the first three waves?

We rewrote it as follows:

*“As explained above in the results section, our hypothesis is that Cluster 3 in wave 4 are elderly people who potentially survived across the first three waves.”*

− In the 2nd paragraph the sentence “The reason is that….those outcomes of COVID-19” is not clear.

We have rearranged the order of the clauses to better emphasize the cause-and-effect relationship between the clustering process and the severity of COVID-19 outcomes in the determined profiles. Thus, the sentences reads as follows:

*“We observed that the COVID-19 patient profiles we identified exhibited varying degrees of COVID-19 outcomes, including in-hospital mortality, ICU assistance, and invasive mechanical ventilation. Interestingly, these outcomes were not used in the process of determining the clusters. We believe this may be because the selection of relevant variables to use in the clustering process was performed using a supervised method based on those COVID-19 outcomes.”*

− In the 2nd sentence of the 3rd paragraph the sentence “were evidenced that did not work” is not correct

Thank you for pointing it out. The new sentences reads as:

*“For example, treatments used in the first wave, such as hydroxychloroquine and lopinavir/ritonavir, were found to be ineffective and were replaced by others like remdesivir and corticosteroids.”*

Conclusions
− The conclusion is brief. It would be nice if the Authors deepened their view about how the clustering approach could help the physicians in a practical setting. For instance, it would be interesting to have some actual examples on how the patient’s management should be differentiated according to the different clustering at hospital admission.

We appreciate your suggestion to provide actual examples on how patient management should be differentiated according to the different clustering at hospital admission. However, we would like to clarify that the conclusion section of our paper follows the requirements of the journal and must be concise. Furthermore, we have already reached the maximum word limit allowed by PlosOne for research work. In addition, our focus was to present the clustering approach as a potential solution to address the heterogeneity of hospitalized-COVID-19 patients. That being said, we agree that it would be valuable to provide more practical examples in future studies. We will certainly keep this suggestion in mind for future work. Once again, thank you for your valuable feedback.

**Reviewer #2**

The article does not present a scientific novelty. It seems only a brief assessment of the Covid 19 waves. Therefore, it is not suitable for publication in the journal.

Thank you for taking the time to review our paper. We respectfully disagree with the reviewer's comment that the paper does not present a scientific novelty and is not suitable for publication in the journal.

It is true that we have not developed a new clustering methodology to identify patient profiles, but the sequential combination of a supervised method (classification tree) to select the most relevant factors, which avoids redundancy, and, especially, the application of an unsupervised semiparametric method (KAMILA) for clustering mixed-type data is a novel contribution to the field. Additionally, we also described in detail the four steps required to implement our proposal (incorporating their references), which makes it more accessible to practitioners and researchers, and, therefore, reproducible.

Furthermore, nowadays, clinical studies have more and more variables collected, and some of them are correlated with each other. Thus, the number of variables is often large for a not very large set of individuals (patients). In these situations, the methods we use are of interest, and this paper is a good example of their application.

Additionally, we used both clinical judgement and supervised statistical methods to reduce the number of covariates and focus on the most informative ones for each wave. Moreover, our study takes into account the effect of COVID-19 vaccines on the last wave, which adds to the relevance of the findings.

In summary, we believe that our paper provides valuable insights and offers practical implications for the application of multiple care intervention strategies, as another way of responding to new outbreaks of this or future diseases.

**Reviewer #3**

Excellent work in collecting and analysing the data.

Thank you very much for your positive feedback on our work. We greatly appreciate your recognition of our efforts in collecting and analysing the data. We believe that our study can contribute to a better understanding of COVID-19 patient profiles and help to improve care intervention strategies.

Consider adding a comparison to other large metropolitan areas in Spain or Europe

Thank you for the suggestion to compare our results with other large metropolitan areas in Spain or Europe. However, we feel that conducting such a comparison would require extensive data collection and analysis beyond the scope of our study, which was already a large multicenter cohort study that spanned four different waves of COVID-19 over a year and a half. Additionally, comparing with other metropolitan areas would require data collection in the same temporal window, which could be difficult to achieve. Therefore, we have decided not to pursue this suggestion in the current manuscript.

We included 3 more references in our study (apart from the already included: Bondeelle et al. (2021), Grant et al. (2020), and Ye et al. (2020)) to related research that has also used patient profiling during the COVID-19 pandemic, incorporating relevant comorbidities, clinical results, and sociodemographic features to examine their relationship with outcomes such as the requirement of IVM, ICU assistance, and in-hospital mortality. These studies, jointly with the previous three already included, provide additional insights into the use of patient profiling in COVID-19, and readers, practitioners, and researchers can compare our results with theirs. We hope that including these references will provide a helpful point of comparison for our readers. The three new references are:

Bondeelle L, Chevret S, Cassonnet S, et al. Profiles and outcomes in patients with COVID-19 admitted to wards of a French oncohematological hospital: A clustering approach. PLoS One. Published online 2021:e0250569-e0250569.

Grant RW, McCloskey J, Hatfield M, et al. Use of latent class analysis and k-means clustering to identify complex patient profiles. JAMA Netw Open. 2020;3(12):e2029068-e2029068.

Ye W, Lu W, Tang Y, et al. Identification of COVID-19 Clinical Phenotypes by Principal Component Analysis-Based Cluster Analysis. Front Med. 2020;7. Accessed September 23, 2022. <https://www.frontiersin.org/articles/10.3389/fmed.2020.570614>

Valladares-Garrido, M. J., Failoc-Rojas, V. E., Soto-Becerra, P., Zeña-Ñañez, S., Torres-Roman, J. S., Fernández-Mogollón, J. L., ... & Díaz-Vélez, C. (2022). Clinical-epidemiologic variation in patients treated in the first and second wave of COVID-19 in Lambayeque, Peru: A cluster analysis. International Journal of Infectious Diseases, 123, 212-220.

<https://www.sciencedirect.com/science/article/pii/S1201971222004428>

Zhou, L., Romero-García, N., Martínez-Miranda, J., Conejero, J. A., García-Gómez, J. M., & Sáez, C. (2022). Subphenotyping of Mexican Patients With COVID-19 at Preadmission To Anticipate Severity Stratification: Age-Sex Unbiased Meta-Clustering Technique. JMIR Public Health and Surveillance, 8(3), e30032.

<https://publichealth.jmir.org/2022/3/e30032/>

Benito-León, J., Del Castillo, M. D., Estirado, A., Ghosh, R., Dubey, S., & Serrano, J. I. (2021). Using unsupervised machine learning to identify age-and sex-independent severity subgroups among patients with COVID-19: observational longitudinal study. Journal of Medical Internet Research, 23(5), e25988.

<https://www.jmir.org/2021/5/e25988/>

Please add a better quality image of Figure 1. Bar plots of the % of the outcome

Thank you for your suggestion to improve the quality of Figure 1. We have updated the figure to include the changes you requested, including adjusting the y-axis scale to be in percentages, adding the percentage values to the barplots, and centering the bars for easier visualisation. We also have the individual plots available if the journal prefers to include them. Please find the revised Figure 1 attached to this response.
