# Supplementary material for: COVID-19 patient profiles over four waves in Barcelona metropolitan area: A clustering approach
Source: PLoS One. 2024 May 7;19(5):e0302461. doi: 10.1371/journal.pone.0302461 (PMC11075871; doi:10.1371/journal.pone.0302461)
Supplement: Attachment — Submitted filename: Response_to_Reviewers_R2.docx [file pone.0302461.s003.docx]

Response to reviewers' comments on the paper “COVID-19 patient profiles over four waves in Barcelona metropolitan area: A clustering approach” – Second Review

March 22^nd^, 2024

**General Comments to Editor**

We sincerely appreciate the chance to submit a revised version of our manuscript for the second round of reviews of our manuscript “*COVID-19 patient profiles over four waves in Barcelona metropolitan area: A clustering approach*” (Manuscript Number: PONE-D-22-31704R1) for PLOS ONE. We carefully considered the reviewers' comments, and below we explain how we have revised the paper based on those comments and recommendations. We hope that these revisions enhance the manuscript's quality and meet the publication standards of PLOS ONE. Below, we offer detailed responses to the reviewers' comments (written in blue), with all changes in the revised manuscript tracked and written in orange to ease their identification. As requested by Reviewer #4, we have included the page and line numbers in the Word file that tracks the changes.

Our revisions focused on the following two key aspects:

- We have enhanced the manuscript by providing additional detail on the methods used for variable selection and cluster analysis. Specifically, we have rewritten sections to offer clearer and more comprehensive explanations of the KAMILA and CART methodologies, as recommended by the reviewer. Our goal in elucidating the KAMILA approach was to improve its clarity and transparency while avoiding unnecessary repetition of foundational concepts.
- We included footnotes to tables to clarify their interpretation.
- As a consequence of extending the description of the KAMILA approach, we have enriched the manuscript by incorporating the following references:

Akhlaghi, M., Tabesh, H., Mahaki, B., Malekpour, M. R., Ghasemi, E., & Mansourian, M. (2022). Predicting the Physician's Specialty Using a Medical Prescription Database. *Computational and Mathematical Methods in Medicine*, *2022*.

Akyea, R. K., Ntaios, G., Kontopantelis, E., Georgiopoulos, G., Soria, D., Asselbergs, F. W., ... & Qureshi, N. (2023). A population-based study exploring phenotypic clusters and clinical outcomes in stroke using unsupervised machine learning approach. *PLOS Digital Health*, *2*(9), e0000334.

Jimeno, J., Roy, M., & Tortora, C. (2021). Clustering mixed-type data: a benchmark study on KAMILA and k-prototypes. In *Data Analysis and Rationality in a Complex World 16* (pp. 83-91). Springer International Publishing.

Perez-Benzo, G. M., Muellers, K., Chen, S., Liu, B., Bagiella, E., O'Conor, R., ... & Federman, A. D. (2021). Identifying behavioral phenotypes in chronic illness: self-management of COPD and comorbid hypertension. *Patient education and counseling*, *104*(3), 627-633.

Preud’Homme, G., Duarte, K., Dalleau, K., Lacomblez, C., Bresso, E., Smaïl-Tabbone, M., ... & Girerd, N. (2021). Head-to-head comparison of clustering methods for heterogeneous data: a simulation-driven benchmark. *Scientific reports*, *11*(1), 4202.

Velasco, V., Gragnano, A., Ghelfi, M., Gruppo Regionale HBSC Lombardia 2014, & Vecchio, L. P. (2023). Health lifestyles during adolescence: clustering of health behaviours and social determinants in Italian adolescents. *Journal of Public Health*, *45*(1), 218-228.

**Response to Reviewer #3**

Congratulations on an excellent work well done. This paper will serve as a guideline for many epidemiologists, not only in Europe but Worldwide

Thank you for your kind words and positive feedback on our work. We greatly appreciate your recognition of the potential impact of our paper on the field of epidemiology. In particular, we believe that our study can contribute to a better understanding of COVID-19 patient profiles and help to improve care intervention strategies.

We have included the following sentence in the revised version of the manuscript (lines 6-7 in page 18):

*We believe that our research has the potential to serve as a guideline for epidemiologists worldwide, extending its applicability beyond Spain.*

**Response to Reviewer #4**

This is a very complex paper describing the application of clustering techniques to help identify groups of patients with COVID-19 who essentially then have differing risks of, for example, death from their disease.

Clustering techniques essentially take variables, ranging from the patient characteristics at presentation, to clinical and laboratory measures. In this study these appear to concern 292 such measures which are then reduced by some means (not fully explained) to 36. In brief, these remaining variables are then exposed to a so-called KAMILA clustering technique which appears (see Table 1 for example) to be reduced to 20 for the Wave 1 group of COVID patients. Sadly, no explanation of how KAMILA works is provided although this is perhaps not so surprising as the reference to Foss and Markatou (2018) describing the technique comprises 43 pages of complex text. Nevertheless, without some explanation of how KAMILA works the reader is left facing a totally ‘black box’ (a total mystery) procedure.

I presume KAMILA somehow reduces the number of variables (here from 36 to 20) and then creates several 20-dimensional clouds of these variables which if distinct (although perhaps overlapping) are then identified as the Clusters. However, whatever this process, the two groups (Wave 1: Cluster 1 & Cluster 2) identified for Table 3 appear to distinguish them by having higher In-hospital mortality, need for ICU assistance, and for Invasive mechanical assistance. Similar findings are reported for Waves 2, 3 and 4.

Personally, I am not very comfortable with the complex processes involved here, but more importantly any non (cluster) technical reader will be mystified by the presentation. I note that the authors all appear to be statisticians (as I am) but I strongly feel that the paper could be much improved with a simple review of the clustering process and some clinical input to reinforce the value of this approach.

Incidentally, this paper without Page and Line numbers included, made reviewing very difficult.

Thank you for taking the time to review our paper. We apologize for any confusion caused by the absence of page and line numbers in the manuscript. We have included them in the Word file that tracks the changes. We acknowledge the complexity of the clustering technique utilized in our study and appreciate your feedback regarding the need for a clearer explanation of the methodology.

As an initial point of feedback, we would like to mention that we employed a utilized supervised non-parametric learning method, specifically a classification tree, to capture complex modeling structures more effectively and select the most relevant variables and, thus, avoiding redundancy in our posterior analysis. Additionally, we performed the KAMILA clustering technique, which is robust for clustering mixed-type data (i.e., categorical and numerical data) in a single procedure. While we understand that the complexity of KAMILA may pose challenges for readers unfamiliar with the technique, we have made efforts to enhance accessibility by providing detailed descriptions of the methodology and the steps required for its implementation. Furthermore, we have included references to support our approach and facilitate reproducibility for practitioners and researchers.

Now, we will proceed to address each specific point raised by the reviewer in detail:

- Selection of the most relevant variables: The initial selection of non-redundant variables was conducted using a classification tree (CART), with a specific focus on discriminating disease outcomes for each wave. To validate this selection, logistic and Cox stepwise regressions with bootstrapping were employed. As a result of this process, the number of variables was successfully reduced from 292 to 36, as detailed in eTable 2 of the Supplement. It is important to note that the KAMILA clustering technique did not influence the selection of variables. Tables 1 and 2 present a comparison of profiles determined by KAMILA, listing only the statistically significant variables identified through Mann-Whitney U tests for numerical variables and Chi-squared tests for categorical variables (totalling 19 variables). Although the variable 'gender' was found to be non-significant, it was included for demographic interest. Consequently, these tables display 20 variables instead of the 36 used in the KAMILA approach.

To clarify this misunderstanding, we have added the following sentence in Tables 1 and 2 (lines 20-21 in page 11 and lines 6-7 in page 12):

*The table only shows the statistically significant variables^1^.*

Additionally, a footnote has been included in the table (lines 1-2 in page 12 and lines 1-2 in page 13):

*1. Based on Mann-Whitney U test for numerical variables and Chi-squared tests for categorical variables. Gender was not significant, but it was included for demographic interest.*

Furthermore, in subsection “2) Guided Variable Selection and Reduction” (lines 20-22 in page 6), we have included the following sentence in the revised version of the manuscript:

*All 36 variables were utilized in the determination of patients’ clusters. However, Tables 1 and 2 only display the 19 statistically significant variables, along with gender for demographic interest, resulting in a total of 20 variables.*

- More comprehensive explanations of the KAMILA: We appreciate the reviewer’s interest in understanding KAMILA in more depth. While we recognize the request for technical specifics, we believe it is appropriate to refer to the original research papers that delve into KAMILA’s development and we have included additional information about the KAMILA procedure, including its initialization and how prediction strengths are evaluated within our manuscript. Furthermore, we have enhanced the manuscript by citing relevant health-related papers where KAMILA has been effectively utilized, which should aid in comprehension.

1. We start including recent studies in the field of health where the KAMILA approach has been applied. These include the following articles:

Akhlaghi, M., Tabesh, H., Mahaki, B., Malekpour, M. R., Ghasemi, E., & Mansourian, M. (2022). Predicting the Physician's Specialty Using a Medical Prescription Database. *Computational and Mathematical Methods in Medicine*, *2022*.

Akyea, R. K., Ntaios, G., Kontopantelis, E., Georgiopoulos, G., Soria, D., Asselbergs, F. W., ... & Qureshi, N. (2023). A population-based study exploring phenotypic clusters and clinical outcomes in stroke using unsupervised machine learning approach. *PLOS Digital Health*, *2*(9), e0000334.

Perez-Benzo, G. M., Muellers, K., Chen, S., Liu, B., Bagiella, E., O'Conor, R., ... & Federman, A. D. (2021). Identifying behavioral phenotypes in chronic illness: self-management of COPD and comorbid hypertension. *Patient education and counseling*, *104*(3), 627-633.

Velasco, V., Gragnano, A., Ghelfi, M., Gruppo Regionale HBSC Lombardia 2014, & Vecchio, L. P. (2023). Health lifestyles during adolescence: clustering of health behaviours and social determinants in Italian adolescents. *Journal of Public Health*, *45*(1), 218-228.

2. KAMILA is well-suited for datasets containing a variety of variable types, including both categorical and numerical variables. Unlike methods like k-means, which rely solely on metric distances, KAMILA offers greater adaptability to different data types, ensuring robust clustering performance. Moreover, KAMILA directly tackles the challenge of balancing continuous and categorical variables in clustering. Its framework facilitates the equitable combination of both types, leading to more accurate and comprehensive clustering analysis^5^. Furthermore, studies have demonstrated KAMILA's superiority in handling high imbalances between continuous and categorical data compared to other methods^6^.
3. From a computational perspective, KAMILA demonstrates superior scalability to larger datasets compared to traditional k-means, making it suitable for big data applications lately encountered in the health field^5^. Furthermore, when compared with other equivalent algorithms, KAMILA exhibits the best performance and is the most time-efficient in dealing with large datasets, particularly those characterized by heterogeneity, as observed in our study^6^.
4. We also considered using alternative clustering methods for mixed-type data, such as k-prototypes. However, the results obtained were equivalent to those found using KAMILA, consistent with the findings of simulation studies^6,7^. For the sake of the article's focus and extension, we have chosen to include only the results of KAMILA.

Based on the considerations mentioned below, we have included the following sentences in the revised version of the manuscript:

- Lines 15-16 in page 7:

*KAMILA has been applied in recent health studies ^1-4^ to stratify individuals into clusters according to the outcomes of interest.*

- Lines 2-4 in page 7:

*Several studies have demonstrated KAMILA's superiority in handling high imbalances between continuous and categorical data compared to other methods^6^.*

- Lines 4-7 in page 7:

*The KAMILA algorithm is a development of the k-means method who combines Gaussian-multinomial mixture model, a model-based approach, with the k-means algorithm, a non-model-based approach, both of which have been adapted successfully to very large data sets.*

- Lines 7-14 in page 7:

*In the KAMILA procedure, each parameter is initialized by randomly drawing from a uniform distribution with bounds set to the minimum and maximum of each continuous variable. Categorical variables are initialized by drawing from a Dirichlet distribution with shape parameters all equal to one. The algorithm is initialized multiple times. For each initialization, the algorithm iterates until either reaching a pre-specified maximum number of iterations or until the population membership remains unchanged from the previous iteration, whichever occurs first.*

- Lines 16-21 in page 8:

*The prediction strength indicates the proportion of pairs of cases classified in the same cluster in both the training and test samples, considering the cluster with the worst performance. Since a separate test sample is often not available, the procedure employs repeated two-fold cross-validation. In this process, the first fold serves as the training sample, while the second fold serves as the test sample.*

**References**

1. Velasco, V., Gragnano, A., Ghelfi, M., Gruppo Regionale HBSC Lombardia 2014, & Vecchio, L. P. (2023). Health lifestyles during adolescence: clustering of health behaviours and social determinants in Italian adolescents. *Journal of Public Health*, *45*(1), 218-228.
2. Perez-Benzo, G. M., Muellers, K., Chen, S., Liu, B., Bagiella, E., O'Conor, R., ... & Federman, A. D. (2021). Identifying behavioral phenotypes in chronic illness: self-management of COPD and comorbid hypertension. *Patient education and counseling*, *104*(3), 627-633.
3. Akhlaghi, M., Tabesh, H., Mahaki, B., Malekpour, M. R., Ghasemi, E., & Mansourian, M. (2022). Predicting the Physician's Specialty Using a Medical Prescription Database. *Computational and Mathematical Methods in Medicine*, *2022*.
4. Akyea, R. K., Ntaios, G., Kontopantelis, E., Georgiopoulos, G., Soria, D., Asselbergs, F. W., ... & Qureshi, N. (2023). A population-based study exploring phenotypic clusters and clinical outcomes in stroke using unsupervised machine learning approach. *PLOS Digital Health*, *2*(9), e0000334.
5. Foss, A., Markatou, M., Ray, B., & Heching, A. (2016). A semiparametric method for clustering mixed data. *Machine Learning*, *105*, 419-458.
6. Preud’Homme, G., Duarte, K., Dalleau, K., Lacomblez, C., Bresso, E., Smaïl-Tabbone, M., ... & Girerd, N. (2021). Head-to-head comparison of clustering methods for heterogeneous data: a simulation-driven benchmark. *Scientific reports*, *11*(1), 4202.
7. Jimeno, J., Roy, M., & Tortora, C. (2021). Clustering mixed-type data: a benchmark study on KAMILA and k-prototypes. In *Data Analysis and Rationality in a Complex World 16* (pp. 83-91). Springer International Publishing.
